# Phytochemical screening and neuro-pharmacological activity of *Mimosa pudica* flowers: Integrating *in vitro*, *in silico* and *in vivo* approaches

**DOI:** 10.1016/j.heliyon.2025.e42017

**Published:** 2025-01-24

**Authors:** Fahmida Alam, Rashedul Alam, A.T.M. Yusuf, Joya Datta Ripa, Raktim Das Nithin, Sourav Barua, Mohammed Fazlul Kabir, Seong-Tshool Hong, Hea-Jong Chung

**Affiliations:** a Department of Pharmacy, University of Science and Technology Chittagong (USTC), Bangladesh; b Department of Biotechnology, Harrisburg University of Science and Technology, United States; c Department of Biomedical Sciences and Institute for Medical Science, Jeonbuk National University Medical School, Jeonju, 54907, South Korea; d Gwanju Center, Korea Basic Science Institute, Gwanju, 61715, South Korea

**Keywords:** Neuro-pharmacology, Molecular docking, GC-MS, *Mimosa pudica*, hMAO, In silico study, Elevated-plus maze, Phytochemical screening

## Abstract

Throughout millennia, medicinal plants have been crucial in preserving human well-being and enhancing the whole human experience.

*Mimosa pudica*, sometimes referred to as the "sensitive plant," possesses considerable promise in the discovery of innovative herbal remedies. The objective of our research is to examine the various pharmacological uses of this mysterious plant by undertaking a thorough investigation of its methanolic extract. We employed sophisticated laboratory techniques to carefully extract and examine the chemical constituents of *M. pudica*. This examination uncovered a wide range of advantageous phytochemicals, such as alkaloids, flavonoids, tannins, and saponins. In order to evaluate the neuro-pharmacological effects of the extract, we conducted a comprehensive set of neurobehavioral tests on Swiss Albino mice. These tests included the open field test, light-dark box test, elevated plus maze test, tail suspension test, forced swim test, Y-maze test, hole cross test, and social interaction test. The extract, given at doses of 200 mg/kg and 400 mg/kg body weight, showed notable effects on neurobehavioral parameters, similar to the conventional medications Diazepam and Escitalopram (1 mg/kg body weight). In addition, we conducted in-silico activities on hMAO A and hMAO B receptors by performing molecular docking studies on 11 compounds that were identified using GC-MS analysis. The results of our investigation revealed the chemical properties of *M. pudica* and emphasized its potential as a substance that can reduce anxiety and depression. It also has effects on memory and learning, which could lead to significant gains in pharmaceutical development and medical progress.

## 1 Introduction

Touch-me-not, *Mimosa pudica*, may cure anxiety and depression. Many cultures have used it for medicinal purposes, and new research reveals that its bioactive constituents may boost GABA action to relax and reduce anxiety [1,2]. Animal tests show it decreases anxiety like diazepam. *M. pudica* controls mood-regulating neurotransmitters like serotonin and dopamine [3,4,5].

Antidepressant benefits are enhanced by its antioxidant content, which lessens oxidative stress, a depression risk. *M. pudica* extracts produce imipramine-like antidepressant effects on rats [6,5].Therapeutic alkaloids, flavonoids, tannins, and steroids are in the plant. Though *M. pudica* is typically harmless, those with medical issues or using medication should consult a doctor before using it. *M. pudica* may cure anxiety and depression naturally in addition to conventional therapies, but most data comes from animal research [7,2].

Worldwide, anxiety disorders enhance worry, fear, and physiological arousal. Depression, on the other hand, causes profound sadness, decreased interest or enjoyment, and sleep, hunger, and cognition problems [8].Depression, on the other hand, causes profound sadness, decreased interest or enjoyment, and sleep, hunger, and cognition problems. *Mimosa* using *M. pudica* to treat anxiety suggests it may be an anxiolytic. Little evidence supports its neuropharmacological anti-anxiety properties. Understanding *M. pudica* flower extract's anxiolytic mechanisms may assist treat anxiety [6].

Depression, on the other hand, causes profound sadness, decreased interest or enjoyment, and sleep, hunger, and cognition problems. *M. pudica* has traditionally been used to boost mood, suggesting it could be an antidepressant [5].

This substance's antidepressant activities are poorly studied, especially in neuropharmacology. The neurochemical pathways altered by *M. pudica* flower extract may disclose its depression-treating processes and help develop new treatments [9]. The recent study found that the ethyl acetate fraction of *M. pudica* (EAMP) may have antidepressant properties due to its flavonoids and alkaloids. It reduced immobility time in mice dose-dependently, similar to established antidepressants, suggesting that norepinephrine and dopamine levels may be modulated [3,10,8].

Cognitive function requires learning and memory for adaptation, problem-solving, and decision-making. Some *M. pudica* phytochemicals may aid memory and learning, according to recent studies. However, few research have identified the cognitive functions *M. pudica* flower extract addresses. Its neuropharmacological effects on memory and learning may disclose new neuroprotection and cognitive enhancement methods [9,11].

Parkinson's disease (PD), the second most common neurodegenerative disease, increases cognitive fragility risk in many patients. PD causes resting tremors, muscle rigidity, and sluggish movement due to midbrain dopamine-producing cell loss [11,7].The flavoenzyme human monoamine oxidase (hMAO) oxidatively deaminates dopamine, a major neurotransmitter, causing PD. HMAO-A and B have different substrate and inhibitor sensitivities [12].

MAOIs were the first antidepressants and remain essential due to major depressive disorder's global health impact. An hMAO-B inhibitor, selegiline prevents dopamine breakdown and regulates Parkinson's motor symptoms. But hMAO-A inhibitors raise serotonin and norepinephrine to MDD. Cognitive fragility and depression benefit from hMAO [13].

In this study, we used in silico molecular docking to test MP-derived phytochemicals against the two hMAO receptor isozymes. Human monoamine oxidase inhibition research relies on molecular docking modeling. This approach predicts the interaction between small molecule inhibitors and the enzyme's active site, revealing binding strength and inhibition potential [14].

In addition to neuro-pharmacological activities, *M. pudica* has wound-healing, antioxidant, anti-inflammatory, and antibacterial properties. Its many bioactivities come from its phytochemical composition of tannins, saponins, phenolic chemicals, alkaloids, and flavonoids [15,3]. The broad pharmacology of *M pudica* reveals its versatility as a medicinal and suggests new treatments for a variety of diseases [16].

Very little neuropharmacology work has been done on *Mimosa pudica*. Especially on flowers. A few studies have shown that the neuro effects are not well established. More research should be done on this.

## 2 Materials and methods

### 2.1 Plant materials

In the course of conducting a thorough scientific study for a research paper, the selection of the plant was meticulously reviewed. Subsequently, the plant flower samples were collected from various locations including the USTC field, Chittagong University field, Foyslake, and Bhatiyari. and authenticated by Professor Dr. Shaikh Bokhtear Uddin, Department of Botany, Chittagong University. The voucher sample was preserved at the Department of Pharmacy, University of Science and Technology Chittagong (USTC).

### 2.2 Preparation of plant extract

The research paper details a scientific methodology employed to extract the methanolic extract from *Mimosa pudica* flowers. It begins with the careful selection of healthy and mature flowers, ensuring optimal vitality and freedom from disease or pest damage. The flowers undergo a thorough cleaning and preparation procedure to remove impurities and extraneous plant parts. The extraction process involves maceration of the plant material in methanol for 14 days at room temperature, followed by filtration to obtain a clear, particulate-free solution. The yield of the methanolic extract is quantified and stored in amber glass vials at −20 °C to maintain stability. Rigorous quality control measures, including safety precautions and adherence to laboratory protocols, are observed throughout the extraction process.

### 2.3 Chemical and drugs

The chemicals and medications utilized in our investigation included Methanol (Merck, Germany), Diazepam 5 mg (Square Pharmaceuticals Ltd., Bangladesh), and Escitalopram 10 mg (Reneta Ltd). The MEMPF was provided orally at doses of 200 and 400 mg/kg, dissolved in distilled water and saline, respectively. The administration took place 30 min prior to the test.

### 2.4 Animals and ethical approval

Swiss albino mice weighing between 20 and 25 g, of both sexes, were obtained from the animal research section of the International Centre for Diarrheal Disease and Research,

Bangladesh (ICDDR, B) is a country. The animals were housed in controlled laboratory circumstances, with a constant room temperature and a relative humidity of 55–65 %. They were exposed to a 12-h light/dark cycle and provided with a regular meal. We adhered to the Ethical Principles and Guidelines for conducting observations on mice, as outlined by Scientific Experiments on Animals. This guideline was authored by the Swiss Academy of Medical Sciences and the Swiss Academy of Science. The Animal Welfare and Husbandry Association, DOP, USTC, Bangladesh has granted approval for our research operations.

### 2.5 Phytochemical screening

The document provides a comprehensive exploration of the phytochemical composition of *Mimosa pudica's* floral extracts. It delves into the qualitative and quantitative analysis of various phytochemical classes, including tannins, phenolic compounds, and flavonoids [17].

The qualitative screening involved the use of classical tests and assays to identify the presence or absence of specific phytochemical classes within the extract [18].

The presence of tannins was confirmed through the Ferric Chloride test, while phenolic compounds were identified by the bluish-green color change upon the addition of ferric chloride solution. Flavonoids were identified through the Shinoda test, which resulted in a bright red color change [7].

The quantitative analysis involved the use of spectrophotometric methods and standard reference compounds to measure the concentrations of total flavonoids, tannins, and phenolic compounds in the floral extract. The results were expressed as a percentage, representing the mass of the standard reference compound equivalent per gram of floral extract. These findings provide valuable insights into the chemical composition of *Mimosa pudica*'s floral extracts and offer a quantitative perspective of the plant's phytochemical richness [17,19].

### 2.6 *In silico* study

#### 2.6.1 Ligand selection

A total of seventy-two substances were obtained from the gas chromatography-mass spectrometry analysis. Following that, a selection of specific compounds, including 4-Methoxy-cyclohexanone, Benzene, nitroso, Benzofuran,2,3-dihydro-,2-Methoxy-4-vinylphenol, Dihydroartemisinin,10,o-(t-butyloxy), 11,13-tetradecadien-1-ol acetate, Cholest-5-ene,3 -methoxy-(3-beta)-, were obtained from the PubChem database pubchem.ncbi.nlm.nih.gov [20].

#### 2.6.2 Assessment of the ligands' suitability as possible therapeutic agents

The physical and molecular characteristics, as well as the pharmacokinetic parameters such as ADMET (absorption, distribution, metabolism, excretion, and toxicity), are crucial factors in determining the suitability of substances as potential therapeutic candidates. The properties of the compounds listed were analyzed using the pKCSM online tool http://biosig.unimelb.edu.au/pkcsm/ to confirm their potential as ligands for therapeutic target [21,20].

The compounds were subsequently subjected to filtration using Lipinski's rule of five in order to forecast their drug likeliness using the SwissADME web server [22].

Out of a total of seventy-two compounds, thirty were deemed eligible for further docking study. The remaining forty-two compounds were eliminated because they violated Lipinski's criteria in more than one parameter [22].

#### 2.6.3 Protein production and identification of the active site

The crystal structures of two target proteins, 2v5z (Safinamide) and 2z5x (Harmine), were obtained from the RCSB protein data bank. The resolution of the structures was determined to be 1.60 Å and 2.20 Å, respectively. The active site of the enzyme was determined after a comprehensive study of existing literature review and docking parameters of standard drugs [23,24].

The required cleaning and preparations, such as removing heteroatoms, cofactors, and water, were performed using BIOVIA Discovery Studio 4.5 Client and Swiss-PdbViewer (v4.1) [23].

The shape was modified by adding hydrogen atoms, and the target protein was then minimized using the MMFF94s force field through the PyRx-virtual screening program. The target protein was stored in pdb format for the purpose of conducting docking experiments [25].

### 2.7 Docking and post-docking

The docking computations were conducted using AutoDock, version 4.2, with the assistance of PyRx 0.3 http://pyrx.scripps.edu. The docking findings were analyzed using PyMOL [24].

These methods can clarify the specific sort of interaction (such as hydrogen-bond, π-π interaction, and cation-π interaction) that played a role in ligand binding. PyMOL was utilized to offer supplementary data regarding the interaction between the ligand and receptor [26].

### 2.8 Statistical research

The mean ± SEM form of the experimental data was used.

The importance of variation among the several handled treatments.

Using one-way ANOVA then Dunnett's multiple comparison tests, groups and control groups were examined. Considered statistically significant was a p 0.05.

## *3* *In-vivo* experimental approach

A total of 54 mice were utilized in the investigation, categorized into separate groups for the assessment of neuropharmacological activity. The groups comprised:

Control group: Six mice were administered saline only as a vehicle control.

Standard group: Six mice received a uniform standard dosage.

Test groups: Three mice were used in each dose group (200 mg/kg and 400 mg/kg). There were a total of 54 mice used for the 8 separate tests that were done in equal amounts.

### 3.1 Open field test

The locomotor abilities of *Mimosa pudica* Flower Extract were evaluated utilizing the open field test. In summary, the mice were introduced to the test room for at least 1 h prior to each test to become accustomed to their surroundings [27].

The open field devices consisted of a Plexiglas square box measuring 50 × 50 × 40 cm, with the floor divided into 25 small squares of equal proportions (10 cm × 10 cm) that were marked by black lines [20].

The test animals in this investigation were randomly allocated into five groups. The control group received a solution of 1 % Tween-80 in saline orally at a dose of 10 ml/kg. The positive control group received Diazepam intraperitoneally at a dose of 1 mg/kg. The treatment groups received MPFE orally at doses of 200 and 400 mg/kg body weight [20].

During a 1-h administration period, each animal was individually positioned at the center of the device and closely monitored for 5 min to quantify the number of squares crossed by the animal using all four of its paws. The open field arena was meticulously sanitized after each test to prevent the animal from being affected by the odors of pee and feces from previous tests(Fig. 2) [27].

**Fig. 1:**
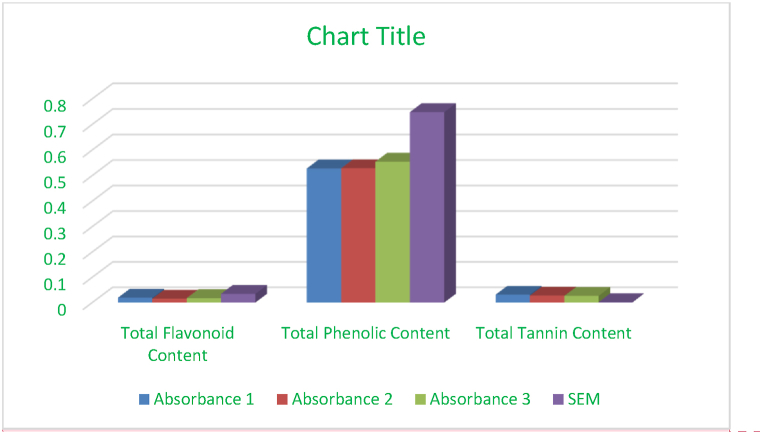
Quantitative comparison among total flavonoids content, total tannin content, total phenol content.

**Fig. 2:**
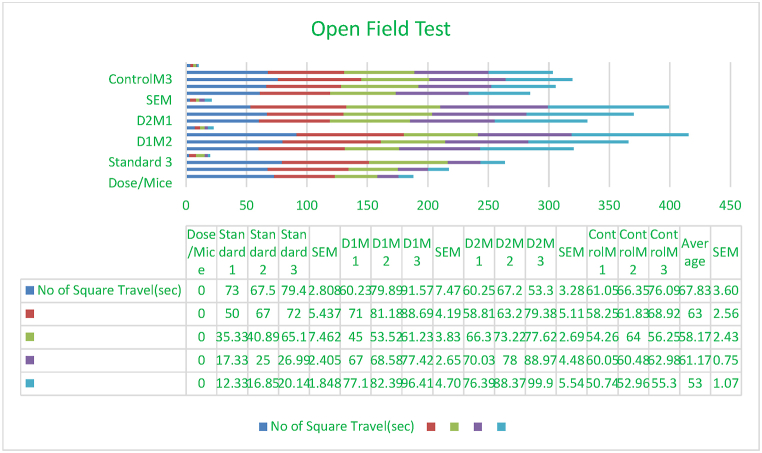
Emphasizing exploratory and sedative effects, analysis of square crossings over time across the treatment groups of 200 mg/kg and 400 mg/kg together with the positive control (diazepam at 1 mg/kg, i.p.).

### 3.2 Hole-cross test

Minor changes were made to Takagi et al.'s (1971) hole cross test method. The mice were randomly assigned to five groups: The control group received 10 ml per kilogram of body weight of 1 % Tween-80 in water orally. Diazepam was given intraperitoneally to the positive control group at 1 mg/kg. The treatment group got 200 and 400 mg per kilogram of body weight oral *Mimosa pudica* flower extract. The animals were then placed in a 30 × 20 × 14 cm3 chamber with a steel wall in the center [28].

The cage's center had a 3 cm diameter, 7.5 cm height hole. After receiving a control, positive control, and various extract concentrations, the animals were allowed to move between chambers through a hole. Animals were counted as they passed through the aperture between chambers. The study captured passages at 0, 30, 60, 90, and 120 min throughout a 5-min period. The open field test formula was used to calculate movement inhibition % (Fig. 8A, Fig. 8BA and B) [29,28].

### 3.3 Elevated plus maze test

With some modifications, Barua CC et al.'s methodology was used. The device features two 5 × 10 cm open arms.

The building has two 5 × 10 × 15 cm arms hanging from a 5 × 5 cm platform. These arms form a plus sign 40 cm above the floor. The open arms had 0.5 cm margins to prevent mice from falling, whereas the closed arms had 15 cm edges [30].

Five animals were in each control, positive control, and test group. The test groups received oral *Mimosa pudica* methanolic extract at 200 and 400 mg/kg body weight. Diazepam was given orally to the positive control group at 1 mg/kg body weight, whereas the control group received 1 % Tween 80 in water at 10 ml/kg. Each animal was placed individually in the center of the Elevated Plus Maze (EPM) and given 5 min to explore after 60 min of test samples. After then, the number of open and closed arm entries and their duration were manually recorded. A soundproof chamber was used for the entire test. Animals entered arms when they placed all four paws on them [31].

Divide the time spent in the open arm by the sum of the time spent in the open arm and the closed arm to get the percentage [31]The percentage of open arm entries is computed by dividing the number of open arm entries by the sum of open arm and closed arm entries(Fig. 4) [31].

### 3.4 Light-dark box test

**Fig. 3:**
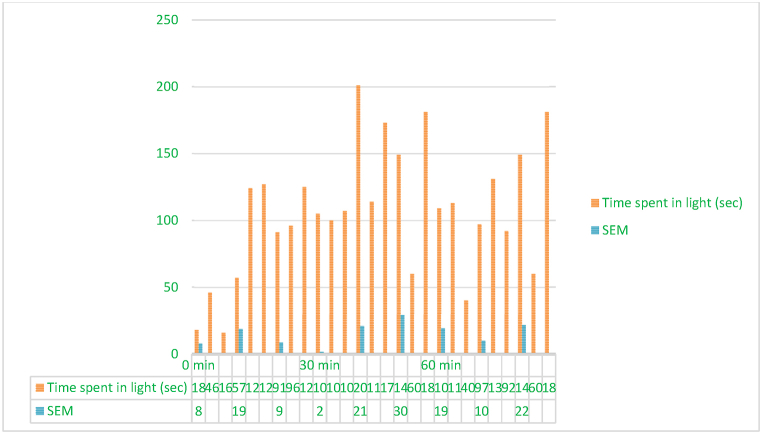
In comparison to the standard anxiolytic control (diazepam at 1 mg/kg, i.p.), the length (in minutes) that mice spent in the light compartment of the light-dark box test after administration of methanolic extract of *Mimosa pudica* flowers at doses of 200 mg/kg and 400 mg/kg.

The equipment consisted of a 45 × 27 × 27 cm rectangular box with two compartments connected by a 7.5 × 7.5 cm wall hole. Mice were observed for 5 min in the middle of the lit area, and their time spent in the white/light part was recorded. The following approach determined the light compartment's time: Multiple the number of seconds spent in the light compartment by 100 and divide by the total number of seconds, 300 s or 5 min of observation time, to calculate the percentage [32] The reference anxiety-reducing drug in the light-dark exploration assay was diazepam (1 mg/kg, intraperitoneally). This choice was taken to set a basis for evaluating, at different dosages, the anxiolytic effectiveness of the plant extract(Fig. 3).

**Fig. 4:**
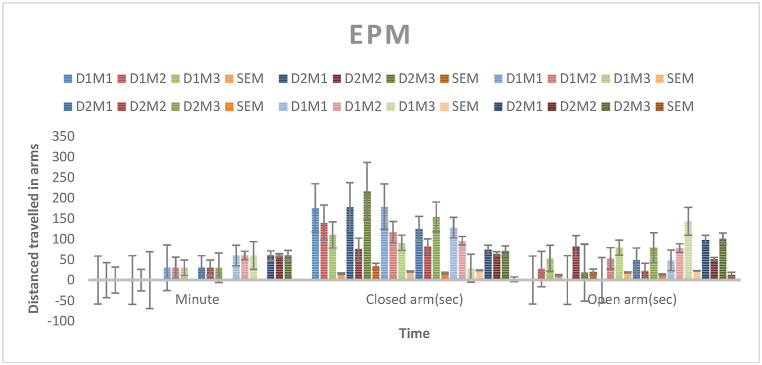
Duration (in seconds) in the open arms of the elevated plus maze by mice administered methanolic extract of *Mimosa pudica* flowers at dosages of 200 mg/kg and 400 mg/kg, in conjunction with the standard anxiolytic control (diazepam at 1 mg/kg, i.p.).

### 3.5 Forced swimming test

Behavioral neuroscience researchers employ the Forced Swimming Test (FST) to measure animal depressive-like behavior [27].

A 10 cm-diameter, 25 cm-high cylindrical container held mice for the experiment. The water depth was 19 cm and the temperature was maintained at 25±1 °C measured the length of immobility for each mouse throughout a 6-min session. Each mouse was considered motionless when it stopped resisting and hung in the water, doing only what was necessary to stay afloat. Reducing immobility time has a therapeutic effect similar to anti-depressants (Fig. 6) [33,27].

### 3.6 Tail suspension test

**Fig. 5:**
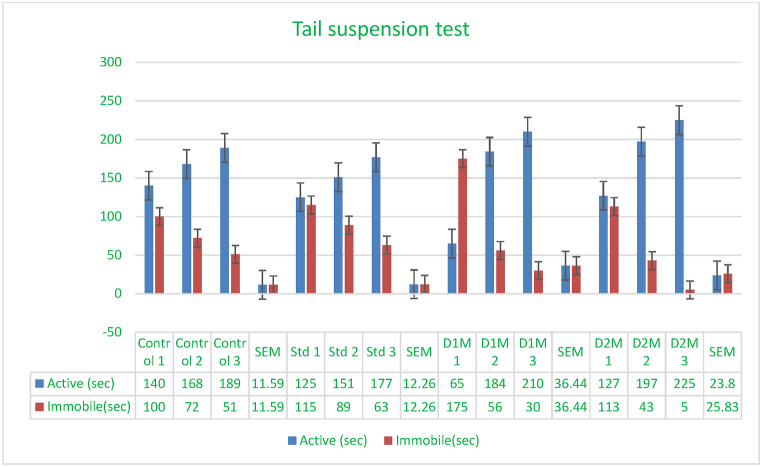
Using escitalopram as the standard control, the tail suspension test measured immobility time (in seconds) for mice administered *Mimosa pudica* methanolic extract at doses of 200 mg/kg and 400 mg/kg.

In the tail suspension test (TST), mice were individually hung by their tails using a metal clamp 10 mm from the tip in a 250 × 250 × 300 mm box, with the head 50 mm above the bottom. The experiment was conducted in a dimly lit room with minimal ambient noise. The mice were hung for a cumulative duration of 6 min, and the period of immobility was measured during the last 4 min(Fig. 5) [34,35].

**Fig. 6:**
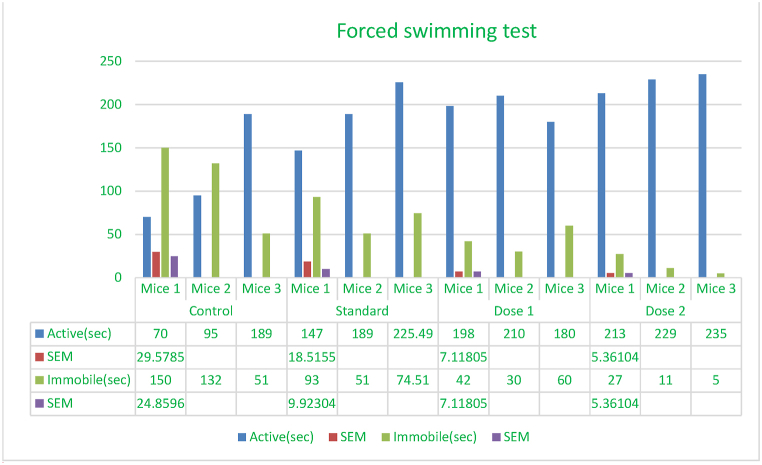
Using escitalopram as the standard control, forced swimming experiments indicate the immobility duration (in seconds) for mice administered with *Mimosa pudica* methanolic extract at dosages of 200 mg/kg and 400 mg/kg.

### 3.7 Social interaction test

A 3-chamber social interaction and novelty apparatus with a core chamber and two similar side chambers was used in the experiment. Each chamber was 30x30cm. Habituation allowed the test subject to get acclimated to the equipment for a defined time. After that, another member of the same species was placed in one side compartment while the other was left empty as a control. Social contact was meticulously monitored during this time. A toy was then placed in one of the adjacent compartments, while a member of the same species inhabited the other. The subject's social novelty was measured by comparing the length of exploring the unfamiliar object to social interaction. This experimental design assessed social interaction behaviors and social novelty preference using the same equipment, revealing participants' social cognition(Fig. 9A, Fig. 9BA and B) [29,36].

**Fig. 7:**
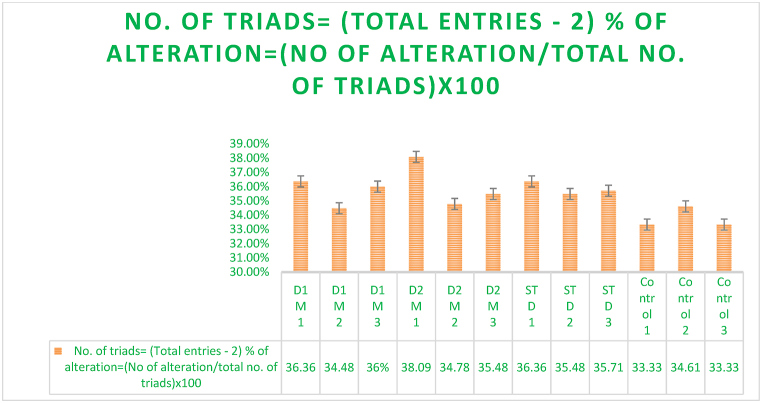
Impact of *Mimosa pudica* methanolic extract at dosages of 200 mg/kg and 400 mg/kg relative to the control group: percentage of spontaneous alternation over time in the Y-maze test.

**Fig. 8A:**
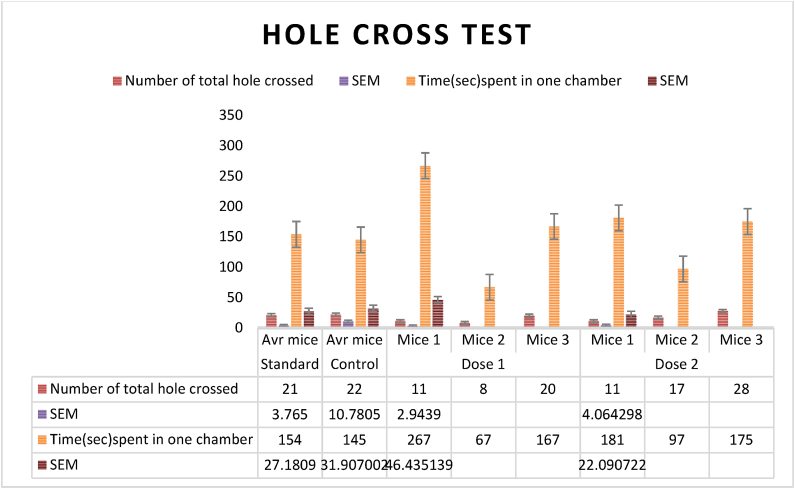
Duration (seconds) spent in a single chamber during the hole-cross test, assessing control, conventional medicine, and *Mimosa pudica* extract (200 mg/kg and 400 mg/kg) and Comparison of the number of holes passed in the hole-cross test among the 200 mg/kg and 400 mg/kg extracts of *Mimosa pudica*, the standard treatment, and the control group.

**Fig. 8B:**
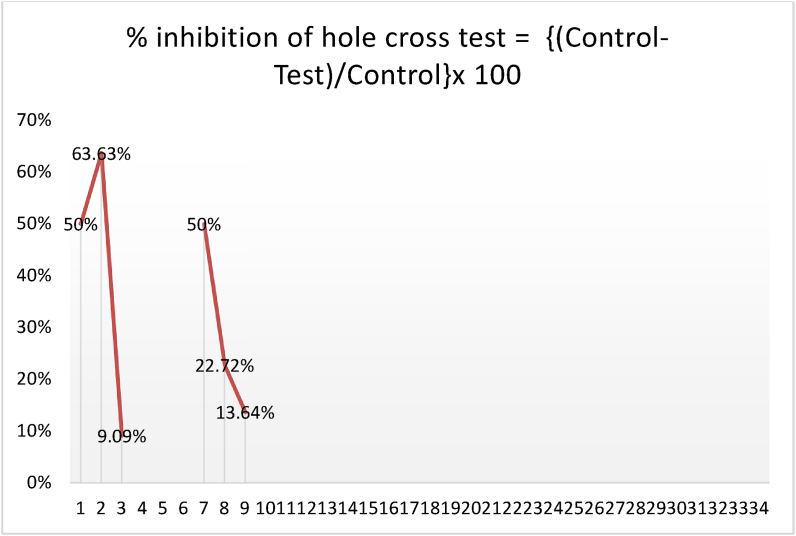
Percentage of Inhibition in the Hole-Cross Test showing dose-dependent effects of *Mimosa pudica* extract.

**Fig. 9A:**
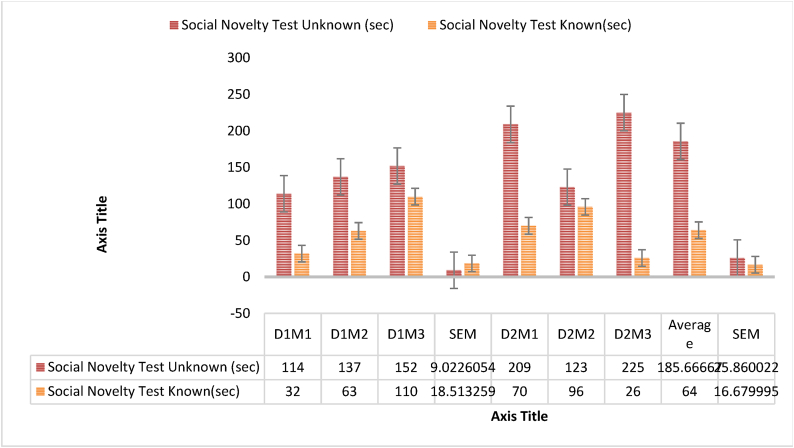
Duration of social interaction (seconds) among treatment groups, comparing control, standard medication, and *Mimosa pudica* extract at dosages of 200 mg/kg and 400 mg/kg.

**Fig. 9B:**
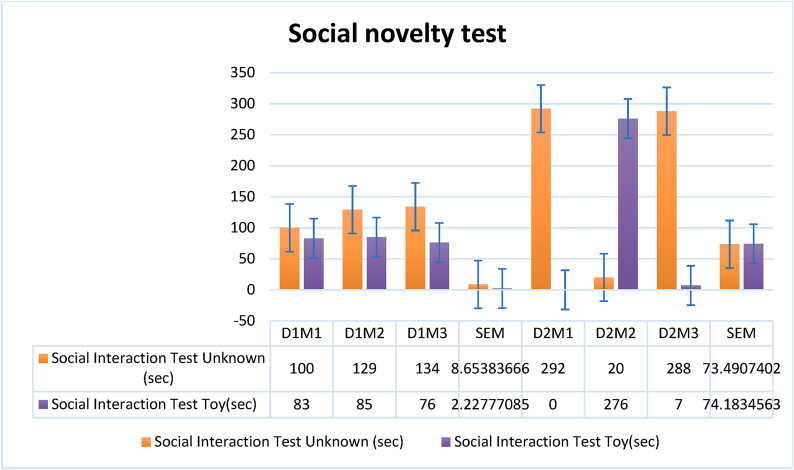
For each group—comprising control, conventional treatment, and dosages of *Mimosa pudica* extract (200 mg/kg and 400 mg/kg)—the duration of social novelty interaction (in seconds) was recorded.

### 3.8 Y maze test

The mouse Y-maze test used three identical arms in a Y form. Each arm was around 30 cm long, 10 cm wide, and 15 cm tall. The trial had a control group and two experimental groups administered 200 mg/kg and 400 mg/kg test drugs. Each group had five mice. Three wooden arms with 120° angles formed the Y-maze. The arms were 30 cm long, 8 cm wide, and 15 cm tall. Every mouse was in the middle of a Y-track.

The exam measured spatial memory and spontaneous change. Each mouse was placed at the end of one maze arm and allowed to explore for 5–10 min. The arm entrance sequence was recorded, and the spontaneous alteration behavior was measured by calculating the percentage of alternations, which are successive entries into three arms. Spatia**l** memory was measured by the frequency of new arm entries [37]. This experimental setup assessed spontaneous alternation behavior and spatial memory ability, revealing the cognitive effects of the test material at different doses (Fig. 7) [38].

## 4 Result

### 4.1 Phytochemical screening

The qualitative analysis verified the existence of significant phytochemical categories, including saponins, tannins, phenolic compounds, and flavonoids, in the plant extract [23]. The quantitative study showed different quantities of various chemicals, with a notably high proportion of phenolic compounds (39 %). This indicates that the plant contains a significant quantity of these potentially beneficial components [39].

### 4.2 Open field test

The open field test was employed to evaluate the exploratory behavior and locomotor activity of mice. The metric quantifies the duration of time spent in the central area of an unobstructed arena, which is indicative of behavior resembling anxiousness [40].

The open field test was utilized to evaluate the motor function, exploratory behavior, and anxiety levels in rats after experiencing cerebral damage and receiving therapy with *Mimosa pudica* extract [40,41].

The study revealed that rats administered with the methanolic extract exhibited less anxiety-like behavior in the open field test as compared to the control groups, suggesting potential anxiolytic effects. Mice administered with the methanolic extract of *Mimosa pudica* exhibited enhanced motor performance and decreased anxiety-like behavior in the open field test, indicating potential neuroprotective properties (Fig. 2) [42].

This study examines the neurological consequences of *Mimosa pudica*, specifically its influence on cognitive function, anxiety levels, and depressive symptoms. The text explores the plant's capacity as a natural treatment for neurodegenerative illnesses and its methods of operation in the central nervous system [43,44].

**Table:** Effect of *M.pudica* extract in Open Field Test on mice

Treatment	Dose(mg/kg)	No of square travels(sec)				
		0 min	30 min	60 min	90 min	120min
Control	0.5ml/mouse	67.83 ± 3.60	63 ± 2.56	58.17 ± 2.43	61.17 ± 0.75	53 ± 1.07
Standard	1 mg/kg b.w	73.3± 2.80	63 ± 5.43	47.10 ± 7.46	23.10 ± 2.40∗∗∗	16.44 ± 1.84∗∗∗
Group I	200 mg/kg	77.23± 7.47	80.29 ± 4.19	53.25 ± 3.83	71 ± 2.65	85.3 ± 4.70∗∗
Group II	400 mg/kg	60.25 ± 3.28	67.13 ± 5.11	72.38 ± 2.69	79 ± 4.48∗	88.22 ± 5.54∗∗

Values are expressed as Mean ± SEM.

∗P < 0.05 compared with the control group (Dunnett's Test).

### 4.3 Light Dark Box test

The Light-Dark box test measures anxiety-like and exploratory behavior in mice. The setup uses a box with two sections: the light chamber is well-lit, while the dark compartment is purposefully dim. An animal in the middle is tracked between the lit and darkened chambers for a set time. Extended time in the dark container decreases anxiety-like behavior, while extended time in the light compartment increases it (Fig. 3) [[44], [45], [46], [47]].

**Table:** Effect of *M.pudica* extract in Light Dark Box on mice

Treatment	Dose(mg/kg)	Time spent in light(sec)		
		0 min	30 min	60 min
Control	0.5ml/mouse	71.28 ± 18.39	62.89 ± 7.23	69.45 ± 12.73
Standard	1 mg/kg b.w	26.66 ± 7.90	104 ± 1.70∗∗∗	87.33 ± 19.34∗
Group I	200 mg/kg	102 ± 18.65	162.66 ± 20.93∗∗∗	106.33 ± 10.003∗∗
Group II	400 mg/kg	104 ± 8.65	130 ± 29.56∗∗	154 ± 21.70∗∗∗

Values are expressed as Mean ± SEM.

∗P < 0.05 compared with the control group (Dunnett's Test).

### 4.4 Elevated plus maze test

This study suggests that *Mimosa pudica* may improve neuroplasticity and anxiety. *Mimosa pudica* may reduce anxiety-like behaviors in the Elevated Plus Maze test via modulating neurotransmitter systems or neurotrophic factors. Our study shows that *Mimosa pudica* reduces stress, promotes neuroplasticity, and improves anxiety. More research is needed to understand the causes and enhance treatment dosage. These preliminary findings suggest that natural compounds like *Mimosa pudica* may be useful for treating psychiatric disorders and increasing brain function(Fig. 4) [[48], [49], [50]].

**Table:** Effect of *M.pudica* extract in Elevated plus maze on mice

Treatment	Dose (mg/kg)	Time spent in close arm and open arm					
		0 min (Close arm)	0 min (Open arm)	30 min (Close arm)	30 min (Open arm)	60 min (Close arm)	60 min (Open arm)
Control	0.5ml/mouse	149 ± 7.11	42 ± 2.32	112 ± 10.54	44 ± 3.12	132 ± 4.90	49 ± 3.45
Standard	1 mg/kg b.w	61.33 ± 14.79	74.33 ± 14.26	77 ± 9.41	78.66 ± 9.68∗	78 ± 13.88	96 ± 13.27∗∗
Group I	200 mg/kg	142 ± 15.57	26.66 ± 12.49	129 ± 21.31	44 ± 18.97	84.33 ± 23.81	89.66 ± 22.89∗∗
Group II	400 mg/kg	157 ± 34.32	33.33 ± 20.31	120.66 ± 17.10	50 ± 13.44	70 ± 2.16∗	83.66 ± 13.36∗∗

Values are expressed as Mean ± SEM.

∗P < 0.05 compared with the control group (Dunnett's Test).

### 4.5 Tail suspension test

This study supports the use of *Mimosa pudica* as an antidepressant. The TST's decrease in immobility time may reduce depressed symptoms by affecting mood-regulating neurotransmitters. More research is needed to understand *Mimosa pudica's* mechanisms and evaluate its long-term effects and safety. *Mimosa pudica's* tail suspension test in rats shows its antidepressant qualities. These findings confirm the historic use of *Mimosa pudica* as a depression remedy. They also suggest greater research to understand its mechanism and clinical efficacy (Fig. 5) [51,33].

**Table:** Effect of *M.pudica* extract in Tail suspension test on mice

Treatment	Dose(mg/kg)	Active and immobile time in 240 s	
		Active(sec)	Inactive(sec)
Control	0.5ml/mouse	165.66 ± 11.58	74.33 ± 11.59
Standard	1 mg/kg b.w	151 ± 12.25	89 ± 12.26
Group I	200 mg/kg	153 ± 36.44	87 ± 36.45
Group II	400 mg/kg	183 ± 23.79∗∗	53.66 ± 25.82

Values are expressed as Mean ± SEM.

∗P < 0.05 compared with the control group (Dunnett's Test).

### 4.6 Forced swimming test

The forced swimming test (FST) is a standard rodent antidepressant effectiveness test. This study tests *Mimosa pudica's* antidepressant potential in rats using the FST paradigm. *Mimosa pudica* extract showed antidepressant-like effects in the forced swim test. The decrease in immobility may modulate depression-related chemicals.

Its antidepressant mechanisms, long-term efficacy, and safety need further study. This study supports *Mimosa pudica* as an antidepressant. This substance's FST model immobility time decrease suggests an antidepressant effect, warranting further exploration of its therapeutic mechanisms and clinical usefulness(Fig. 6) [[52], [53], [54]].

**Table:** Effect of *M.pudica* extract in Forced swimming test on mice

Treatment	Dose(mg/kg)	Active and immobile time in 240 s	
		Active(sec)	Inactive(sec)
Control	0.5ml/mouse	118 ± 29.57	111 ± 24.85
Standard	1 mg/kg b.w	187.16 ± 18.51∗∗	72.83 ± 9.92
Group I	200 mg/kg	196 ± 7.11∗∗	44 ± 7.12
Group II	400 mg/kg	225.66 ± 5.36∗∗∗	14.33 ± 5.36

Values are expressed as Mean ± SEM.

∗P < 0.05 compared with the control group (Dunnett's Test).

### 4.7 Y maze test

The cognitive abilities of *Mimosa pudica* plants were tested in Y-mazes. The plants' movement across the Y-maze's start arm and two alternative arms was recorded. The plants showed a significant preference for one of the alternate pathways, indicating their ability to make informed decisions based on environmental information [55].

This choice suggests that *Mimosa pudica* has fundamental spatial memory and decision-making abilities, supporting the idea that it has cognitive abilities beyond reflexive reactions. It also suggests that environmental cues including light intensity, humidity, and chemical gradients may cause this behavior. To better comprehend *Mimosa pudica's* neurological and physiological processes that affect cognition, the text emphasizes controlled environmental study and molecular analysis (Prieur & Jadavji, 2019). This suggests that the Y-maze test is a good framework for studying *Mimosa pudica's* cognitive capacities. It suggests adding behavioral studies to better understand the plant's cognitive range. The document concludes that *Mimosa pudica* possesses cognitive capacities beyond thigmotactic reactions. It shows that additional research is needed to comprehend this plant species' complicated cognitive processes and ecological and evolutionary value(Fig. 7) [37,29].

### 4.8 The hole cross test

**Table:** Effect of *M.pudica* extract in Y-Maze Test on mice

Treatment	Dose(mg/kg)	% of alteration
		% of alteration= (No of alteration/total no. of triads)x100
Control	0.5ml/mouse	36 ± 5.43
Standard	1 mg/kg b.w	36.12 ± 12.22
Group I	200 mg/kg	35.85 ± 24.09∗
Group II	400 mg/kg	33.76 ± 31.80∗∗

Values are expressed as Mean ± SEM.

∗P < 0.05 compared with the control group (Dunnett's Test).

### 4.9 Hole cross test

The hole cross test tracks *Mimosa pudica* plant behavior. It uses a grid with evenly spaced holes. The researchers recorded the frequency with which the plant traversed the apertures during a certain period and found significant differences in exploratory inclinations among species. Some plants explored extensively, whereas others did not. The statistical study found a link between light intensity, humidity, and plant exploration [56]. According to the study, plants may be more or less curious about their environment. Genetics and physiological processes may explain these inequalities in future studies. Environmental variables affect exploratory behavior, hence behavioral studies should consider the plant's habitat and growing conditions. The study shows that animal behavioral tests can be used to study plant behavior, revealing fundamental plant behavior and ecology. These findings affect agriculture, ecology, and plant neurobiology(Fig. 8A,8B) [56,57].

% inhibition of hole crossed test: {(Control-test)/control}x100.

**Table:** Effect of *M.pudica* extract in Hole Cross Test on mice

Treatment	Dose(mg/kg)	Number of crossing hole and spent time in one chamber	
		Number of holes crossed	Time(sec) Spent in one chamber
Control	0.5ml/mouse	21 ± 2.12	132 ± 10.45
Standard	1 mg/kg b.w	8 ± 1.17	168 ± 19.34
Group I	200 mg/kg	13 ± 2.94∗	166 ± 46.43
Group II	400 mg/kg	18 ± 4.06	151 ± 22.09

Values are expressed as Mean ± SEM.

∗P < 0.05 compared with the control group (Dunnett's Test).

### 4.10 Social interaction and social novelty test

The experiment involved a social interaction test conducted on *Mimosa pudica* plants. The study found that when the plants were alone, they showed very little reaction to touch. However, when they were near one other, the responded to touch in a coordinated way, suggesting the existence of social relationships. The intensity of social contact was shown to be influenced by factors such as plant density and closeness. Higher densities and closer proximity led to more frequent and prominent synchronized responses [58]. The discovered social interaction patterns indicate the existence of intricate communication mechanisms within the species, possibly developed as a means of survival. The study underscores the ecological importance of plant communication and social dynamics, emphasizing the potential benefits in distributing resources, protecting against herbivores, and adjusting to changes in the environment. The results emphasize the significance of taking into account the actions of plants and their social interactions in ecosystem functioning. This highlights the need for additional research to understand the physiological and molecular mechanisms that drive social behavior in *Mimosa pudica* (Fig. 9A, Fig. 9BA and B) [58,59].

**Table:** Effect of *M.pudica* extract in Social Novelty test and Social interaction test on mice

Treatment	Dose(mg/kg)	Novelty test with unknown mice and toy on dose induced mice	
		Unknown	Toy
Group I	200 mg/kg	134 ± 9.02	68.33 ± 18.51
Group II	400 mg/kg	185.66 ± 25.86	64 ± 16.67
Treatment	Dose(mg/kg)	Interaction test on dose induced mice with unknown mice and known mice	
		Unknown	Known
Group I	200 mg/kg	121 ± 8.65	81.33 ± 2.22
Group II	400 mg/kg	200 ± 73.49	94.33 ± 74.18

Values are expressed as Mean ± SEM.

∗P < 0.05 compared with the control group (Dunnett's Test).

### 4.11 Gas chromatography-mass spectroscopy

GC-MS analysis found alkaloids, flavonoids, terpenoids, and phenolic substances in *Mimosa pudica* extract. *Mimosa pudica's* phytochemical molecules were identified using retention time and mass spectrum analysis. Bioactive components of *Mimosa pudica* can be identified by GC-MS phytochemical analysis [60].

These chemicals can treat depression alone or together. Further research is needed to isolate, define, and assess substances' pharmacological characteristics. Gas chromatography-mass spectrometry was used to characterize *Mimosa pudica's* phytochemistry. Bioactive substances make it a promising therapeutic natural product source. These chemicals' medicinal mechanisms and clinical uses need further study [60].

### 4.12 *In-silico* study on potential compound

#### 4.12.1 *In silico* ADMET and drug-likeness studies

*In silico* ADMET analysis is a part of screening potential compounds by using web-based tools so that their therapeutic efficiency can be calculated. In our study, we have used the compounds from GC-MS data obtained from methanolic extract of *M. pudica.* We have investigated the pharmacokinetic profiling such as Blood brain barrier (BBB) permeability, bioavailability, drug likeness, toxicity, and other parameters of the potential compounds before the molecular docking analysis. The phytochemical profiling of our investigated phytochemicals is depicted in Table 1. We examined around seventy-two compounds isolated from this plant and found that these compounds presented in the table, have potential drug likeness parameters. A few compounds could not overcome the blood brain barrier parameter but showed their other criteria matched the criteria of an effective ligands. That is why, in our study, we considered both the BBB permeable and non-permeable compounds for identifying their potential binding affinities. Moreover, for phytochemical screening, we have used SwissADME (drug likeness), pKCSM,(ADME) and ProTox 3 (toxicity study) online servers for investigating different parameters of the phytochemicals. Notably, most of the compounds presented in this table showed impressive phytochemical profile during this study. The compounds that could not follow the cut-off point of Lipniski rule of 5 (Yes/Yes(1) violation), was not considered for further docking analysis.

**Table 1:** Qualitative analysis result.

Phytochemical Constituent	Test Performed	Result
Cardenolides	Kedde's test	Absent
Alkaloids	Dragendorff's test & Mayer's test	Absent
Flavonoids	Shinoda test	Positive
Saponins	Froth Test	Positive
Tannin	Ferric Chloride test	Positive
Phenols	Ferric Chloride Test	Positive

### 4.13 Molecular docking study, post-docking analysis and docking validation

Molecular docking study implements identification of possible connection between receptor and ligands by observing the docking score and non-bonding interactions. During this study, docking score and non-bond interaction of two standard drug with the selected protein is taken as a reference for evaluating the binding patterns of the natural compounds. For our docking purposes, we have used the compounds of which have a moderate drug likeness property according to the ADMET analysis. Here, we have studied two isozymes of human mono amine oxidase receptors (h-MAOA) and (h-MAO B) for finding the anti-depression activities of our compounds as well as two identify the selectivity of the compounds among these two enzymes.

In our study, docking score and non-bonding interactions of selected phytochemicals, with standard drug (Diazepam) that have been used in *In-vivo* studies was investigated. For validation of our docking studies, we have used the docking parameter analysis of the bounded ligands named Harmine and Safinamide which are the co-crystallized standard drugs of human monoamine oxidase A (PDB:2Z5X) and Human Monoamine Oxidase B (PDB:2V5Z).

Biovia Discovery Studio Visualizer Program was used for identifying the binding affinities among the best docked molecule and amino acid residues of the targeted proteins shown in Fig. 1, Fig. 2, Fig. 3, Fig. 4. According to our study, our best docked compound, Benzamide, N-[5-(4-pyridinyl)-1H-1,2,4-triazol-3-yl]- had the similar binding mode like the h-MAO B receptor inhibitor safinamide and our standard drug diazepam (Table 2and Fig. 1, Fig. 3). Interestingly, the docking score of this compound was more than Safinamide (−9.5 kcal/mol) and Diazepam (−9.1 kcal/mol). Besides, the non-bond interactions also showed that this compound possessed impressive binding affinity with both receptors. In h-MAO B binding cavity, it formed strong pi-sulfur bond with CYS A:172, pi-sigma bond with ILE A:199, LEU A:171 and TYR A:326 residues with the shortest distance of intermolecular bindings. As these types of bonds plays the crucial role of drug-receptor interaction with human mono amine oxidases, these bonds with shorter intermolecular length (approximately below 5 Å) ultimately creates strong binding affinity and higher binding score. Additionally, out of the total 72 compounds, 2-Formyl-9-[.beta.-d-ribofuranosyl] hypoxanthine had impressive binding activities against both of the isozymes (−7.7 kcal/mol). Benzofuran,2,3-dihydro- had lowest binding score against both of the enzymes (−6.3 kcal/mol for h-MAO A and −6.1 for h-MAO B).According to the binding affinity, the highest docking tendency was observed against the h-MAO B receptors and also the standard drug showed relatively higher binding affinity towards the isozyme with −9.1 kcal/mol binding affinity. Therefore, the binding affinity result and docking scores of the compounds indicates the selectivity of the compounds against the h-MAO B isozyme over the h-MAO A. Fig. 5 portrays the validity of standard drug safinamide superimposition with h-MAO B receptor (see Table 3) (see Fig. 11.B (a), Fig. 11.B (b), Fig. 11.A).

**Table 2:** Summary of an open field test performed with methanolic extract of *Mimosa pudica* flowers at dosages of 200 mg/kg and 400 mg/kg, intended to investigate it's potential anxiolytic and neuroprotective effects.

Research	Open Field Test (OFT)	Methanolic Extract
Anxiolytic Study	Reduced anxiety-like behavior; indicated by increased time spent in center of arena	Prepared using standard extraction procedures
Neuroprotective Study	Improved motor function and reduced anxiety-like behavior; indicative of neuroprotective effects	Administered orally to rats at specified doses

**Table 3:** GC-MS analysis revealing significant findings from 82 compounds in the methanolic extract of *M.pudica* flowers.

2-Furanmethanol	L-Proline, 5-oxo-, methyl ester
2-Methyl-3-(methylthio) -1-propene	6-Deoxy-D-mannono-4-lactone
Triethylenediamine (TEDA)	1,2,3-Benzenetriol (pyrogallol)
Monomethyl malonate	2-Methoxy-4-vinylphenol (eugenol)

**Fig. 10:**
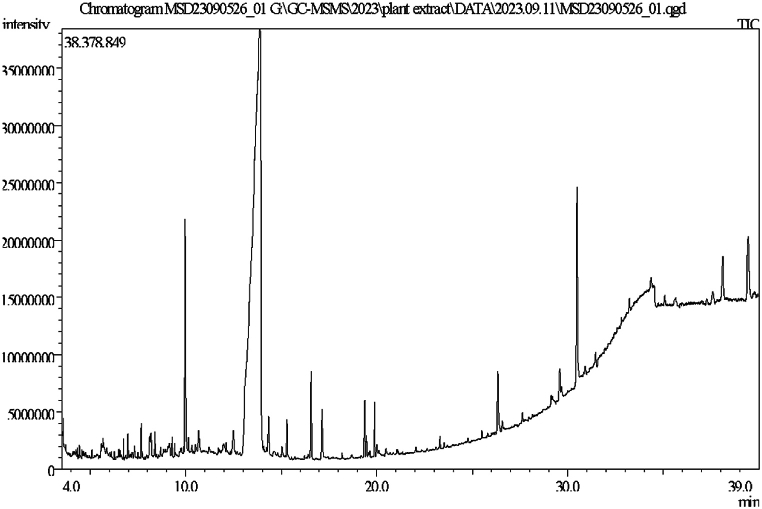
Chromatogram showing GC-MS analytical results for *Mimosa pudica* flowers extracted methodologically.

**Fig. 11.A:**
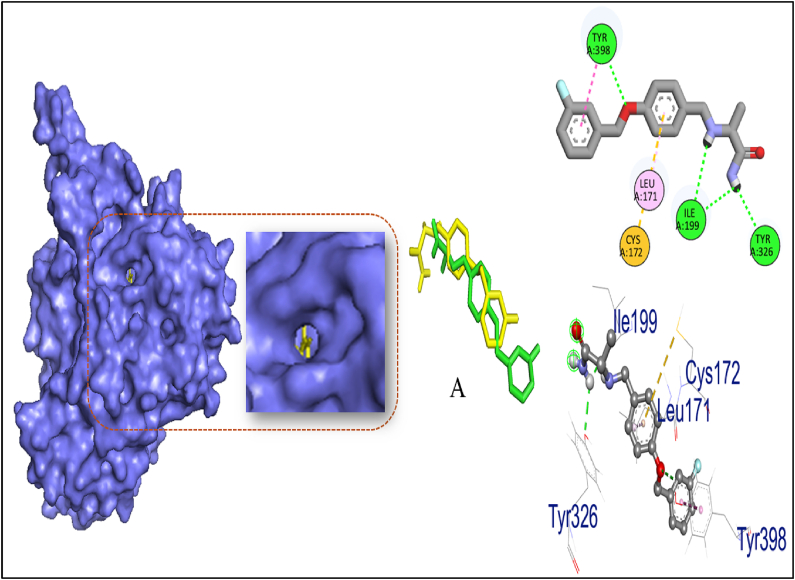
Molecular docking of h-MAO B receptor with standard compound safinamide and non-bond interaction analysis.

**Fig. 11.B (a):**
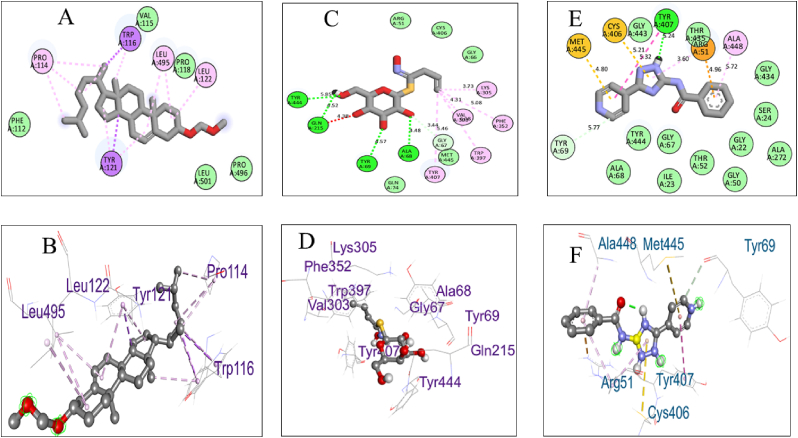
Pictorial representation of the compounds 5 (A and B), 4 (C and D), and 1 (E and F) binding sites showing the interaction between the inhibitors and catalytic active sites of hMAO-A.

**Fig. 11.B (b):**
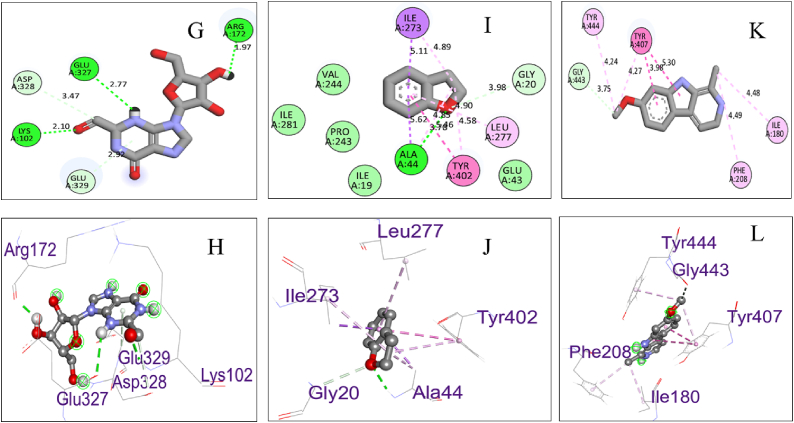
Pictorial representation of the compounds 2 (G and H), 6 (I and J), and 7 (K and L) binding sites showing the interaction between the inhibitors and catalytic active sites of hMAO-A.

#### 4.13.1 Docking validation by re-docking analysis

In order to validate our molecular docking method we have considered re docking analysis of co-crystallized ligands (1). Here, we implemented molecular docking of safinamide which is a selective inhibitor of human MAO B and co-crystalized ligand with PDB ID: 2V5Z. Similarly, for human MAO A we have used harmine which is a beta-carboline alkaloid and a co-crystallized ligand of 2Z5X receptor. We retrieved the structural data of this compounds from PuBChem database (Safinamide, Compound CID: 131682) and (Harmine, Compound CID: 5280953). After that we conducted molecular docking with the same grid box set-up that we considered for our compounds docking. After docking, the non-bond interaction were analyzed by Discovery Studio Software and ChimeraX software. Afterwards, we considered superimposition point analysis were also done to know whether the docking has been done appropriately in the same pocket. The results are shown in the following Fig. 12.A (a), Fig. 12.A (b), Fig. 12.A (c)(a) and (b), (c) [61].

**Fig. 12.A (a):**
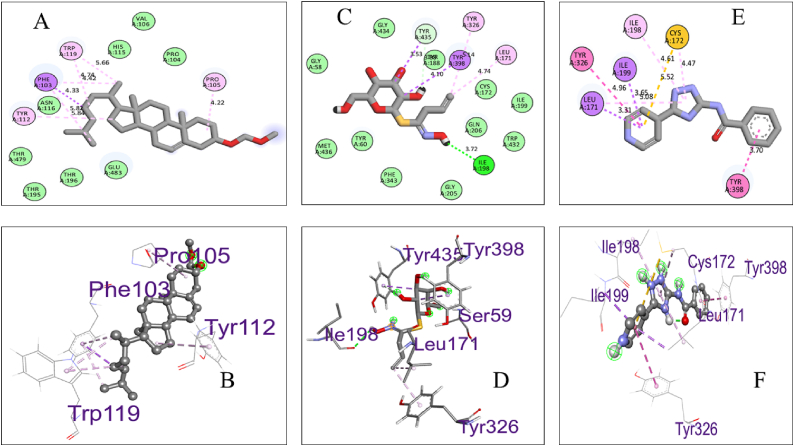
Pictorial representation of the compounds 5 (A and B), 4 (C and D), and 1 (E and F) binding sites showing the interaction between the inhibitors and catalytic active sites of hMAO-B.

**Fig. 12.A (b):**
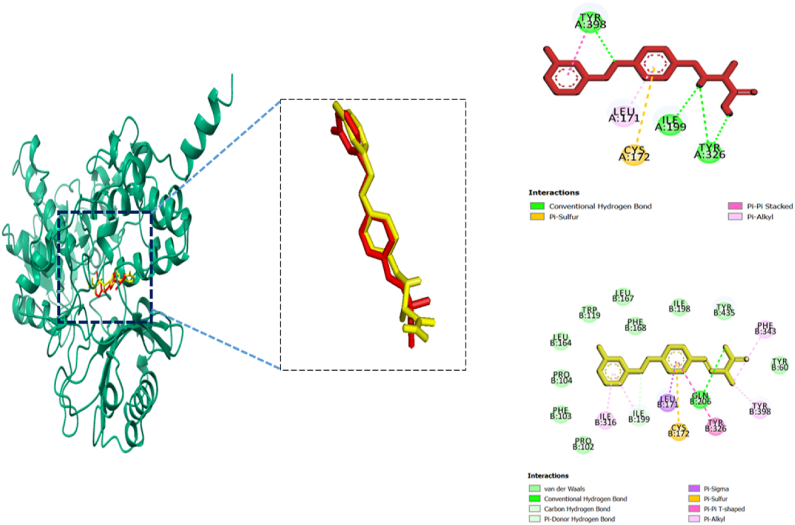
Validation of docking result by redocking analysis with the cocrystal ligand (safinamide) with h-MAO B receptor. The conformation of each cocrystal ligands is displayed in a green stick while docked pose is illustrated in yellow stick. The non-bond interaction studies showed similarity in the binding modes.

**Fig. 12.A (c):**
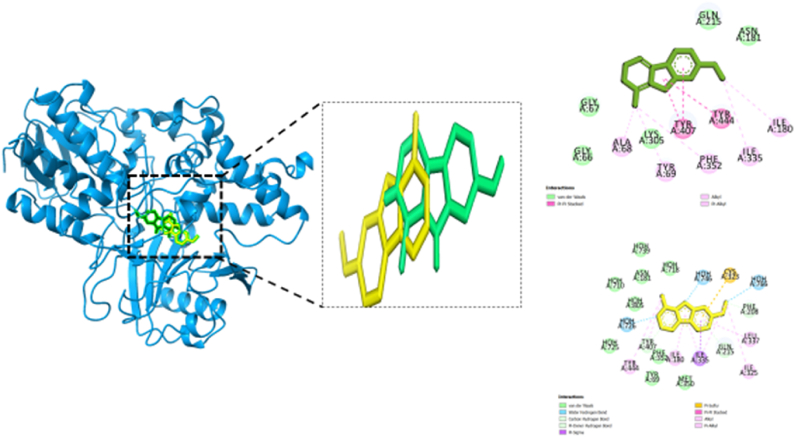
Validation of docking result by redocking analysis with the co-crystal ligand (safinamide) with h-MAO B receptor. The conformation of co-crystal ligand is displayed in a green stick while docked pose is illustrated in yellow stick. The non-bond interaction studies showed similarity in the binding modes.

### 4.14 Discussion

Utilizing in silico ADME/T and molecular docking research is an effective approach for discovering novel pharmaceuticals to treat a diverse array of ailments. This technique offers the benefit of being more time-efficient and cost-effective compared to standard laboratory tests [62,63]. The pharmacokinetic characteristics and drug-likeliness of the phytochemicals found in *Mimosa pudica* were assessed, and the majority of the compounds exhibited significant outcomes [62]. Out of the group, thirty compounds successfully cleared the drug likeness assessment without any signs of toxicity. This research employed a docking method that utilized open software programs and modeled interactions between the ligands 4-Methoxy-cyclohexanone, Benzamide, N-[5-(4-pyridinyl)-1A-1,2,4-triazol-3-yl], and 2-formyl-9-[beta-d-ribofuranosyl]. The compounds hypoxanthine, cholest-5-ene,3-methoxy-(3-beta), etc., interact with the enzymes safinamide and harmine. A crucial step in ligand docking involves the establishment of non-bonding contacts, including hydrogen bonds, hydrophobic interactions, and van der Waals interactions, with vital amino acids. These interactions are identified by the docking score (Fig. 10) [21,64].

Our research indicates that the interaction between ligands and proteins involves a significant number of amino acid residues, particularly in hydrophobic and van der Waals bond interactions. The compound Benzamide, N-[5-(4-pyridinyl)-1H-1,2,4-triazol-3-yl]-, 2-Formyl-9-[.beta.-d-ribofuranosyl]hypoxanthine, and the compound Cholest-5-ene, 3beta-(methoxymethoxy)-, exhibited the highest docking score. This score was similar to the docking scores of the standard drugs Escitalopram and Diclofenac. Furthermore, these compounds displayed excellent non-bonding interactions with the Harmine and Safinamide enzymes. It is important to mention that ligands 4-Methoxy-cyclohexanone, Benzamide, N-[5-(4-pyridinyl)-1A-1,2,4-triazol-3-yl], and 2-formyl-9-[beta-d-ribofuranosyl] have exceptional pharmacokinetic properties and have successfully cleared a drug likeness screening test. These data provide additional evidence that they may be potent chemicals for reducing anxiety and depression and stabilizing mood. This is particularly true in terms of their ability to inhibit hMAO A and hMAO B and their other neuropharmacological effects (Table 4, Table 5).

**Table 4:** *In silico* ADME/T and drug likeliness profiling of potential compounds extracted from *M.pudica* flower.

Compound's name	Absorption		Distribution		Metabolism	Excretion	Toxicity		Drug likeliness	Bio-availability
	Water solubility (log mol/L)	Intestinal Absorption (human)(% Ab-sorbed)	VDss (Human) (log l/kg)	BBB permeability (Log BB)	CYP3A4 substrate	Total clearance (log ml/min/kg)	Neuro toxicity	Hepatotoxicity		
Benzene, nitroso	−1.936	94.085	0.118	Yes	No	0.119	Active 0.53	Inactive 0.61	Yes	0.55
4-Methoxy-cyclohexanone	−0.627	100	−0.034	Yes	No	0.558	Active 0.51	Inactive 0.70	Yes	0.55
Benzofuran,2,3-dihydro-	−1.594	96.548	0.195	Yes	No	0.224	Active 0.57	Inactive 0.76	Yes	0.55
2-Methoxy-4-vinylphenol	−1.853	93.481	0.401	Yes	No	0.239	Active 0.54	Inactive 0.66	Yes	0.55
Dihydroartemisinin,10,o-(t-butyloxy)	−4.608	94.944	0.444	Yes	No	0.769	In-active 0.83	Inactive 0.79	Yes	0.55
11,13-tetradecadien-1-ol acetate	−6.139	93.585	0.317	Yes	No	1.93	In-active 0.76	Inactive 0.76	Yes	0.55
2-formyl-9-[beta-d-ribofuranosyl]hypoxanthene	−2.086	45.256	−0.408	No	No	0.745	Active 0.66	Inactive 0.65	Yes	0.55
Benzamide,N-[5-(4-pyridinyl)-1A-1,2,4-triazol-3-yl]	−3.032	97.82	0.439	No	No	0.03	Active 0.66	Inactive 0.65	Yes	0.55
Desulphosinigrin	−1.591	40.096	−0.217	No	No	0.658	Inactive 0.78	Inactive 0.67	Yes	0.55
Methyl 14-methyl-eicosanoate	−7.907	90.0	0.291	No	No	1.951	Inactive 0.80	Inactive 0.58	No(1V)	0.55
Cholest-5-ene,3 -methoxy-(3-beta)-	−6.834	95.766	0.202	No	No	0.624	Active 0.51	Inactive 0.84	No(1V)	0.55

**Table 5:** Docking score and non-bonding interaction of selected compounds with target receptors hMAO A and hMAO B.

hMAO A 2z5x				hMAO B 2v5z		
SL No	Selected Compounds	Docking Score (Kcal/mol)	Interaction	Selected Compounds	Docking Score	Interaction (Kcal/mol)
1	Benzamide, N-[5-(4-pyridinyl)-1H-1,2,4-triazol-3-yl]-	−9.6	ALA A:448, MET A:445, TYR A:69, ARG A:51, TYR A: 406	Benzamide, N-[5-(4-pyridinyl)-1H-1,2,4-triazol-3-yl]-	−10.3	ILE A:198, ILE A: 199, CYS A:172, TYR A:398, LEU A:171, TYR A: 326
2	2-Formyl-9-[.beta.-d-ribofuranosyl]hypoxanthine	−7.7	ARG A:172, GLU A:327,GLU A:329, ASP A: 328,LYS A:102	2-Formyl-9-[.beta.-d-ribofuranosyl]hypoxanthine	−7.7	PHE A:103,THR A:478,GLU A: 483, ASN A:116
4	Desulphosinigrin	−6.7	GLY A:67,ALA A:68,TYR A:69,GLN A:215,LYS A:305,PHE A:352,VAL A:303,TRP A:397,TYR A:407,TYR A:444	Desulphosinigrin	−7.2	TYR A:435, TYR A:398, SER A:59, LEU A:171, TYR A:326, ILE A:198
5	Cholest-5-ene, 3beta-(methoxymethoxy)-	−7.6	LEU A:122, PRO A:114, TYR A:121, TRP A:116, LEU A:495	Cholest-5-ene, 3beta-(methoxymethoxy)-	−7.3	PRO A:105, PHE A:103, TYR A:112, TRP A:119
6	Benzofuran,2,3-dihydro-	−6.3	LEU A: 277, ILE A: 273,GLY A:20,ALA A:44, TYR A:402,LEU A: 277	Benzofuran,2,3-dihydro-	−6.1	TYR A:435,TYR A:398
7	Harmine	−7.9	TYR A:435,TYR A:398, SER A:59,LEU A:171,TYR A:326,ILE A:198	Safinamide	−9.5	ILE A:198,ILE A:199,CYS:172,LEU A:172,TYR A:398,TYR A:328
8	Diazepam	−6.1	LEU A:176,ARG A: 172,GLU A:329,GLU A:185	Diazepam	−9.1	TYR A:398, PHE A:343, VAL A:294,GLY A:57,GLY A:58,CYS A:397,TRP A:388,MET A:436,ARG A:42

Significant p-values confirmed the dose-dependent neuropharmacological effects of *Mimosa pudica* extract demonstrated by the behavioral tests. The 400 mg/kg dosage in the open field test (Fig. 2) clearly increased locomotion at 120 min (88.22 ± 5.54, **p < 0.01**), therefore indicating enhanced exploratory behavior. The 400 mg/kg dosage significantly increased the time spent in the light compartment (154 ± 21.70, **p < 0.001**) in the light-dark box test (Fig. 3), therefore confirming anxiolytic effects. Although sedative effects were more prominent at 400 mg/kg, the 200 mg/kg dosage greatly extended the time spent in the open arms at 60 min (89.66 ± 22.89, **p < 0.01**) in the elevated plus maze test (Fig. 4). With the 400 mg/kg dose greatly lowering immobility (53.66 ± 25.82, **p < 0.01**), the tail suspension test (Fig. 5) revealed antidepressant-like effects. These findings were corroborated by the forced swimming test (Fig. 6); the 400 mg/kg dosage showed the largest significant effect (14.33 ± 5.36, **p < 0.001**). Significant improvements in crossings for both 200 mg/kg (13 ± 2.94, **p < 0.05**) and 400 mg/kg (18 ± 4.06) revealed less anxiety-like behavior indicated by the hole-cross test (Fig. 8A, Fig. 8BA and B). The 400 mg/kg dosage produced increased exploratory activity and an enhanced interaction duration with unfamiliar mice (185.66 ± 25.86) in social interaction and novelty tests (Fig. 9A, Fig. 9BA and B). Lastly, in the Y-maze test (Fig. 7), no appreciable changes in percentage alternation were seen (**p > 0.05**), most likely because of sedative effects compromising memory capacity at high dosages. Resolving the reviewers' issues about statistical significance, the results—supported by statistical studies—showcase the possible anxiolytic, antidepressant, and sedative effects of the extract.

However, dose-dependent activity variations and calming effects overshadowing memory performance suggest more research. Using molecular docking, metabolomics, and receptor-binding assays, future studies should target neurotransmitter pathways like the GABAergic and serotonergic systems to determine the mechanisms of action. Further behavioral models and pharmacokinetic and pharmacodynamic investigations are needed to determine the extract's efficacy and safety. As a motivated researcher, I want to use cutting-edge methods to better comprehend *Mimosa pudica's* therapeutic potential and develop it into a pharmaceutical drug.

## 5 Conclusion

The results of our investigation indicate that many Phyto-constituents found in MEMP demonstrate antidepressant effects, improve memory and learning, and have anti-anxiety properties. These findings indicate that the MEMP has comparable pharmacological effects to diazepam and escitalopram, with some instances of greater efficacy observed when modifying the dosage.

However, additional in-depth research is necessary to fully understand the precise mechanism underlying the sedative effects and to pinpoint the specific bio-active compound(s) responsible for the reported activity in animal behavioral models.

## CRediT authorship contribution statement

**Fahmida Alam:** Writing – review & editing, Writing – original draft, Resources, Data curation. **Rashedul Alam:** Resources, Investigation, Funding acquisition, Conceptualization. **A.T.M. Yusuf:** Supervision, Project administration, Methodology, Investigation, Data curation, Conceptualization. **Joya Datta Ripa:** Software, Methodology, Formal analysis, Data curation, Conceptualization. **Raktim Das Nithin:** Resources, Methodology, Data curation, Conceptualization. **Sourav Barua:** Visualization, Investigation, Data curation. **Mohammed Fazlul Kabir:** Investigation, Funding acquisition, Formal analysis. **Seong-Tshool Hong:** Resources, Investigation, Funding acquisition, Formal analysis. **Hea-Jong Chung:** Visualization, Validation, Supervision, Funding acquisition.

## Declaration of competing interest

The authors declare the following financial interests/personal relationships which may be considered as potential competing interests: ATM Yusuf reports financial support, article publishing charges, and writing assistance were provided by Gwanju Center, 10.13039/501100003716Korea Basic Science Institute. ATM Yusuf reports a relationship with Gwanju Center, 10.13039/501100003716Korea Basic Science Institute that includes: funding grants and non-financial support. If there are other authors, they declare that they have no known competing financial interests or personal relationships that could have appeared to influence the work reported in this paper.
